# On Combining Reference Data to Improve Imputation Accuracy

**DOI:** 10.1371/journal.pone.0055600

**Published:** 2013-01-30

**Authors:** Jun Chen, Ji-Gang Zhang, Jian Li, Yu-Fang Pei, Hong-Wen Deng

**Affiliations:** 1 Center for Bioinformatics and Genomics, Department of Biostatistics and Bioinformatics, School of Public Health and Tropical Medicine, Tulane University, New Orleans, Louisiana, United States of America; 2 Center of System Biomedical Sciences, University of Shanghai for Science and Technology, Shanghai, P. R. China; 3 College of Life Sciences and Bioengineering, Beijing Jiaotong University, Beijing, P. R. China; University of California, Irvine, United States of America

## Abstract

Genotype imputation is an important tool in human genetics studies, which uses reference sets with known genotypes and prior knowledge on linkage disequilibrium and recombination rates to infer un-typed alleles for human genetic variations at a low cost. The reference sets used by current imputation approaches are based on HapMap data, and/or based on recently available next-generation sequencing (NGS) data such as data generated by the 1000 Genomes Project. However, with different coverage and call rates for different NGS data sets, how to integrate NGS data sets of different accuracy as well as previously available reference data as references in imputation is not an easy task and has not been systematically investigated. In this study, we performed a comprehensive assessment of three strategies on using NGS data and previously available reference data in genotype imputation for both simulated data and empirical data, in order to obtain guidelines for optimal reference set construction. Briefly, we considered three strategies: strategy 1 uses one NGS data as a reference; strategy 2 imputes samples by using multiple individual data sets of different accuracy as independent references and then combines the imputed samples with samples based on the high accuracy reference selected when overlapping occurs; and strategy 3 combines multiple available data sets as a single reference after imputing each other. We used three software (MACH, IMPUTE2 and BEAGLE) for assessing the performances of these three strategies. Our results show that strategy 2 and strategy 3 have higher imputation accuracy than strategy 1. Particularly, strategy 2 is the best strategy across all the conditions that we have investigated, producing the best accuracy of imputation for rare variant. Our study is helpful in guiding application of imputation methods in next generation association analyses.

## Introduction

Genotype imputation, using references with known genotypes and prior knowledge on linkage disequilibrium and recombination rates to infer un-typed alleles for human genetic variations at a low cost, plays an important role in genome-wide association studies (GWAS). Imputation helps to increase both the number of SNPs and power of detection for GWAS. It also allows researchers to combine experiments carried out on different platforms (e.g. Illumina and Affymetrix arrays) for meta-analyses, replication and comparison of finding across studies [1]–[5]. The reference sets used by imputation approaches can be different types such as GWAS data and/or next-generation sequencing (NGS) data.

HapMap as reference sets has been used for most previous imputation studies. With the development of NGS technologies, using NGS data as reference sets for imputation provides comprehensive coverage for the genetic variants, as NGS technologies have the potential to discover the entire spectrum of sequence variations [6]: over 15 million SNPs have been recorded by the 1000 Genomes Project [7], and the number of datasets are increasing day by day. Various researches on imputation and relevant usage of reference sets have been conducted [1], [8], [9], showing that the imputation accuracy increased with increasing sample sizes for the reference sets. Wang et al. [10] created a new reference set with common and uncommon SNPs using existing reference to improve imputation accuracy. Li et al. [11] utilized high depth resequencing data for exons and flanking regions to increase the performance of genotype imputation for rare variants compared to traditional GWAS reference panel. The Oxford-GSK study used 1000 Genomes data as reference for imputation to refine a single genetic region and successfully identified a SNP with a more significant *P*-value than that without 1000 Genomes imputation [12]. The Sardiana study utilized the reference panels from HapMap2, HapMap3 [13], and 1000 Genomes Project [7] separately for the same set of GWAS data [14]. Despite of these efforts, how to efficiently and effectively integrate recently generated NGS data along with previously available references for imputation is still a challenging task, as different NGS data sets may have different call rates and coverage, making it difficult to directly combine data. NGS technologies may also have higher missing rates than the conventional sequencing methods [15]–[18]. Thus, it is important to systematically investigate how to best use multiple reference data to improve genotype imputation accuracy.

In this study, we evaluated three strategies on using NGS data and previously available references (e.g., HapMap data) for imputation. We focused our investigation on two types of data: one has low genotyping accuracy with high marker density (denoted as “R1”), and the other high genotyping accuracy with low marker density (“R2”). The strategies included:

Strategy 1: performing imputation using R1 as reference;

Strategy 2: performing two imputation analyses using R1 and R2, respectively, as reference, and combining the imputed results with results based on R2 selected when overlapping occurs;

Strategy 3: performing imputation using a combined reference based on R1 and R2. The combined reference was obtained through a two-step procedure: 1) imputed R2 by using R1 as reference, and 2) combined R1 and the imputed results from R2.

For investigating the performance of these three strategies, we used both simulated and empirical data. We evaluated three factors affecting imputation accuracy, including LD level, MAF, and marker density. We selected three commonly used imputation methods: MACH [19], IMPUTE2 [20], and BEAGLE [2], [21], all of which have successfully improved power in association analyses [5], [20], [22]–[24]. The results showed that strategy 2 has produced the best imputation accuracy across all the conditions that we have investigated.

## Materials and Methods

### Simulated data

To mimic the LD patterns of the observed human populations, we simulated our data based on HapMap3 [13] and 1000 Genomes datasets [7]. We selected these two datasets for two reasons. One is that they were the most popular reference sets used for imputation, and the other is that they provide a contrast in terms of genomic coverage and data accuracy. For example, the HapMap data were based on direct genotyping from previously discovered SNPs and have been scrutinized thoroughly, and were expected to be of high accuracy. On the other hand, the currently available 1000 Genomes datasets were based on low depth whole genome sequencing data, and thus were regarded to be of lower accuracy. Three segments of the chromosome 22 were selected for subsequent analyses, ranging from 2.1 Mb to 4.2 Mb in length, and with average recombination rates of 4.10 cM/Mb, 1.87 cM/Mb, and 0.85 cM/Mb, corresponding to regions with low, medium and high levels of LD. The simulations were performed in the software package HAPGEN2 [25], [26]. Ten replications were performed for each condition. Briefly, the simulation process included two steps:

Step 1: Sample and variant selection. We randomly selected eighty CEU (Utah Residents with Northern and Western European ancestry) individuals shared by HapMap3 project and 1000 Genomes project. The genetic variant data for the same individual from different projects were combined as follows: when genetic variants were observed in both projects, those from HapMap3 were used; and variants observed in just one project were kept as they were.

Step 2: Simulated sample generation, and reference and validation sample determination. Based on the samples and genetic variants selected in Step 1, we generated the phased haplotype samples of 250 individuals using HAPGEN2. Among these 250 individuals, fifty were selected as validation samples. For these validation samples, a proportion of SNPs were randomly selected as having known genotypes, and the remaining SNPs were considered un-typed and their genotypes would be inferred through imputation. The remaining 200 individuals simulated by HAPGEN2 were randomly put into two groups, each with 100 samples, as reference populations. The first reference population (R1) had its markers generated based on the 1000 Genomes marker map to mimic a high marker density population. It also had 2% randomly simulated error rate for its markers genotypes. The second reference population (R2) had its markers generated based on HapMap marker map, mimicking the low marker density populations. It had a randomly simulated error rate of 1% for its genotypes.

### Empirical data

Phased haplotype data for CEU samples were downloaded from the HapMap3 and 1000 Genomes websites. To obtain the validation samples, we randomly chose 30 common individuals between HapMap3 and 1000 Genomes projects. The remaining individuals, 83 in the HapMap3 and 253 in the 1000 Genomes were assigned into the HapMap3 reference samples and 1000 Genomes reference samples, respectively. To make the coordinate consistency, we transferred the built 36 coordinates to build 37 coordinates through the UCSC LiftOver algorithm [27]. The same three chromosomal regions on chromosome 22 used for the simulated data were selected. In the validation samples, different proportions of markers were masked, for studying imputation accuracy. The analyses were then conducted for each of 10 replications, and imputation accuracy was reported.

### Measures of imputation accuracy

Imputation accuracy was evaluated by two metrics: allele error rate and rare variant error rate.

#### Allele error rate (AER)

AER measures the proportion of incorrectly imputed alleles, which was calculated as the total number of errors divided by twice the total number of imputed loci (the product of the number of individuals and the number of loci). The number of errors was counted as 0 when the imputed and observed marker genotypes were identical, 1 if the real marker genotype was homozygous and the imputed genotype was heterozygous (or vice versa), and 2 if real and imputed marker genotypes were opposite homozygotes.

#### Rare variant error rate (RVER)

RVER was calculated as the total number of rare variant errors divided by the number of rare variants, in consideration that most of rare variant association analyses were based on rare variant number counts. The number of errors was counted as 0 when the imputed rare variant and observed variant types were identical, 1 if the real and imputed marker type were opposite. All the homozygous sites and heterozygous sites for rare variants were considered in the study.

## Results

### Analyses of simulated data

**Figure 1:**
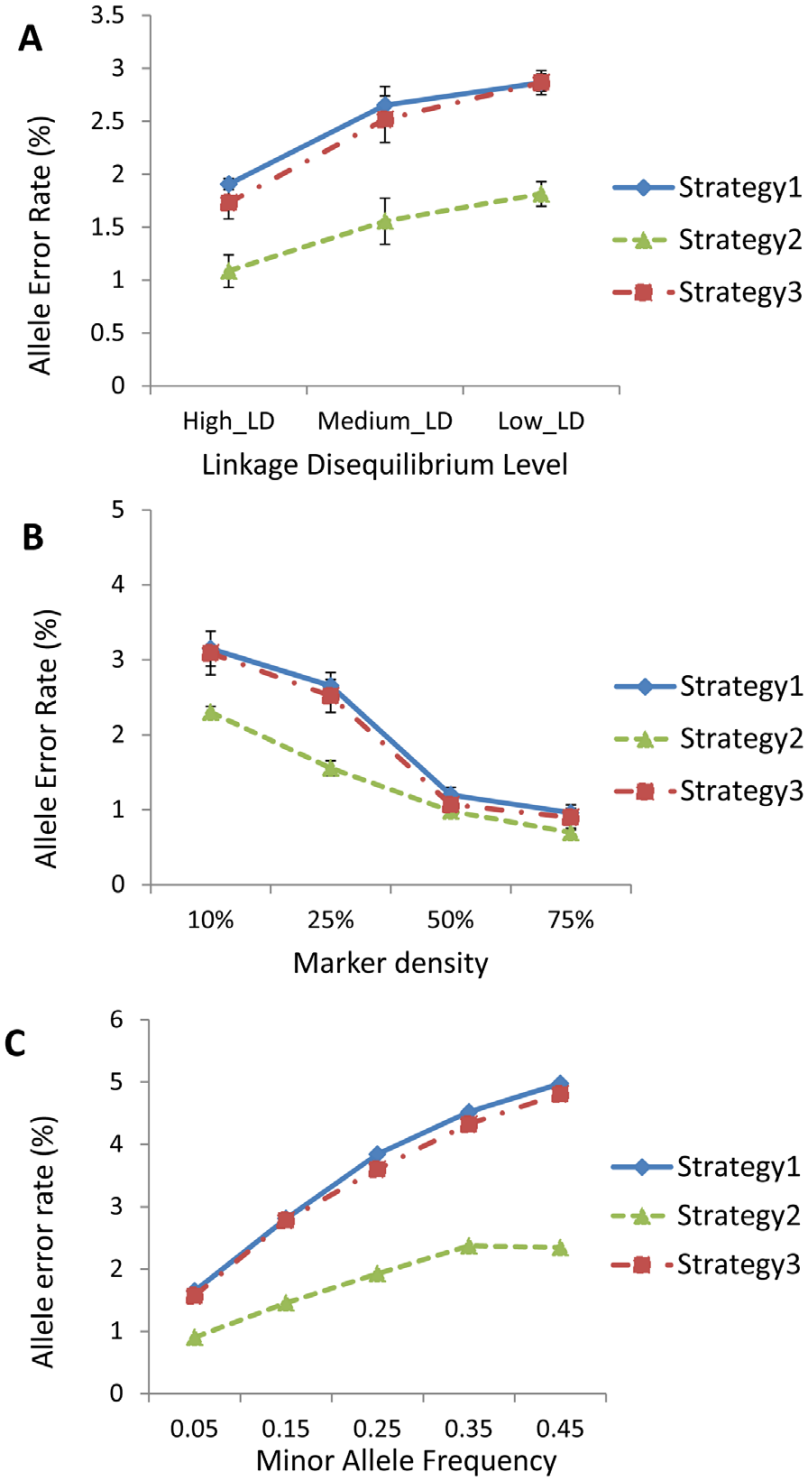
Effects of various factors on allele error rates (AERs). The results are obtained through the software MACH using simulated datasets. (a) Effects of LD levels on AERs, under 25% marker density. (b) Effects of marker density, under medium LD level. (c) Effects of MAF bin of un-genotyped SNPs, under 25% marker density and medium LD level.

We first assessed the performance of the three strategies under different LD levels (results shown in Figure 1A). Under every strategy, AERs under decreased remarkably as LD became stronger. For example, when the LD increased from low to high, AERs for MACH decreased from 2.87% to 1.91% for strategy 1, from 1.81% to 1.09% for strategy 2, and from 2.86% to 1.73% for strategy 3, respectively. Similar trends were seen for the other two imputation methods (Figure S1). Overall, strategy 2 yielded lowest AERs among three strategies, and both strategy 2 and strategy 3 produced lower AERs than strategy 1 under all LD levels simulated, indicating the performance advantages by strategies 2 and 3 over strategy 1.

The effects of marker density on AERs were shown in Figure 1B. As expected, higher density of typed markers led to better performances for imputation. For strategy 1, under the medium LD level, the AER of MACH was 3.15% for 10% marker density (one SNP per 10 SNPs). When the marker density increased to 25%, 50%, and 75%, the AERs decreased to 2.65%, 1.20%, and 0.96%, respectively. For strategy 2, the AERs decreased from 2.30% to 0.69%, and the AERs decreased from 3.09% to 0.89% for the strategy 3, when the marker density increased from 10% to 75%. Similar trends were also observed for the other imputation methods. Overall, AERs attained by strategy 2 and by strategy 3 were similar under higher marker density, but for low marker density, AERs of strategy 2 was 26.91% lower under 10% marker density and 41.34% lower under 25% marker density than those of strategy 3. For all marker densities, strategy 2 and strategy 3 yielded lower AERs than strategy 1 did, again indicating the better performance of strategies 2 and 3 than strategy 1.

Figure 1C showed the impacts of different MAF levels on imputation accuracy for three strategies. In general, AERs increased as MAFs of un-genotyped markers increased for any combination of strategy and imputation software used. For example, when the MAF interval increased from 0.05 to 0.45, AERs for MACH increased from 1.65% to 4.98%, from 0.90% to 2.35%, and from 1.57% to 4.81%, under strategy 1, strategy 2, and strategy 3 respectively. When the imputation accuracy was compared across different strategies, superior results were usually obtained by strategy 2, relative to those for strategy 1 and strategy 3; and similar results were obtained for strategy 3 and strategy 1. The extent of superiority by strategy 2 varied with different MAF. (See Table S1, S2, S3 for the detailed estimates used in Figure 1).

**Figure 2:**
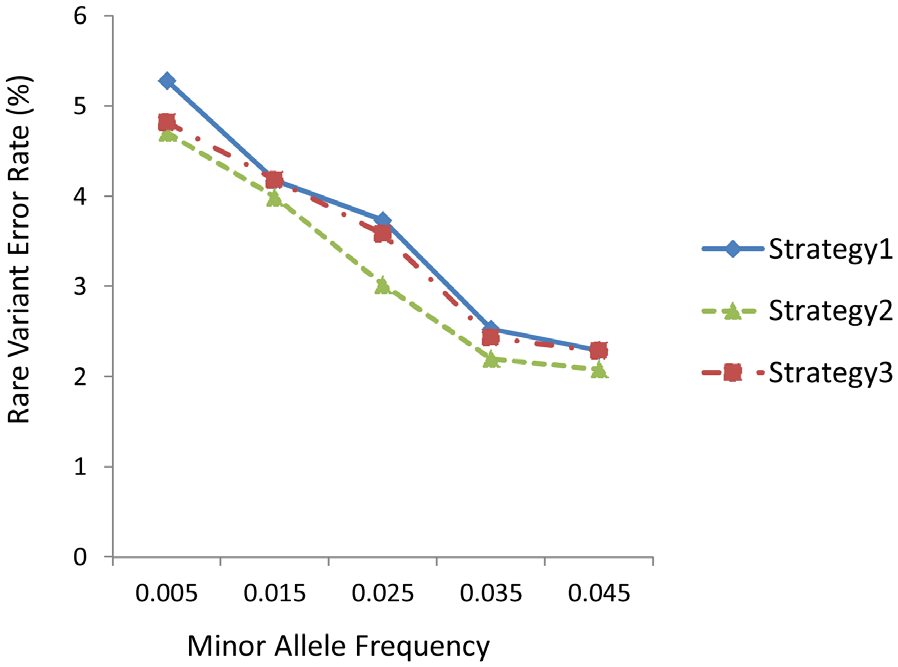
Influence of low frequency rare variant bin on rare variant error rates, under medium LD level and 25% marker density. The results are obtained through the software MACH using simulated data.

The imputation accuracy for rare variants, measured by rare variant error rates (RVERs), under different situations were shown in Figure 2. In general, RVERs decreased as MAF of un-genotyped rare variants increased under medium LD level for all three strategies. For example, when the MAF interval increased from 0.50×10^−2^ to 4.50×10^−2^, RVERs for MACH decreased from 5.28% to 2.29%, from 4.71% to 2.08%, and from 4.82% to 2.28%, for strategy 1, strategy 2, and strategy 3, respectively. Similar trends were also observed for the other LD levels and/or imputation methods. For between strategy comparisons, RVERs for strategy 2 were on average 8.22% and 11.33%, respectively, lower than those of strategy 3 and those of strategy 1 across the MAF spectrum. Strategy 1 and strategy 3 were similar in terms of RVERs at the higher minor allele frequency, and at 0.50×10^−2^ of MAF, strategy 3 had 8.71% lower RVERs than those of strategy 1.

### Analyses of empirical data

**Figure 3:**
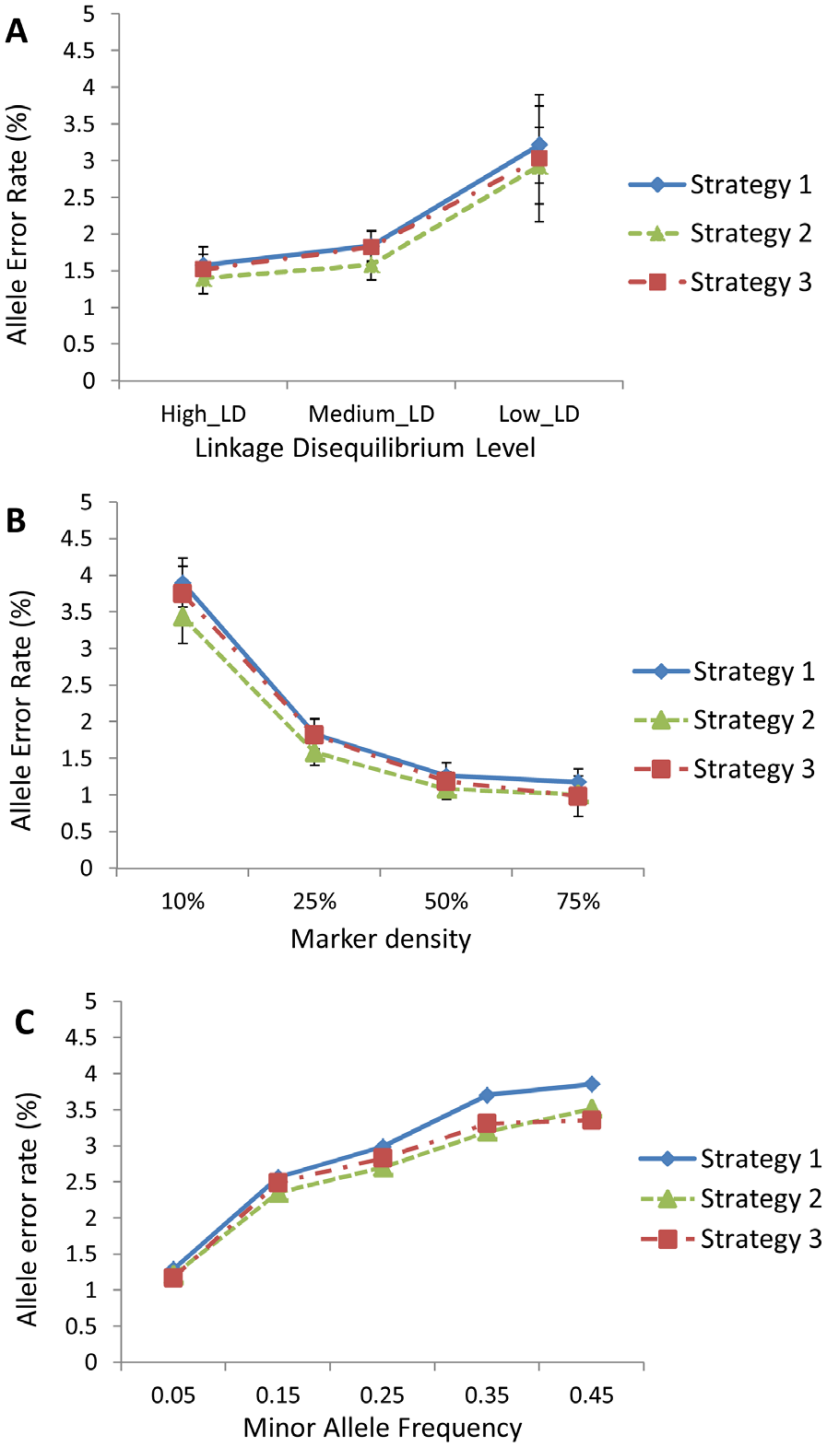
Effects of various factors on allele error rates. The results are obtained through the software MACH using empirical datasets. (a) Effects of LD level on AERs, under 25% marker density; (b) Effects of marker density on AERs, under medium LD level. (c) Effects of MAF bin of un-genotyped SNPs on AERs, under 25% marker density and medium LD level.

**Figure 4:**
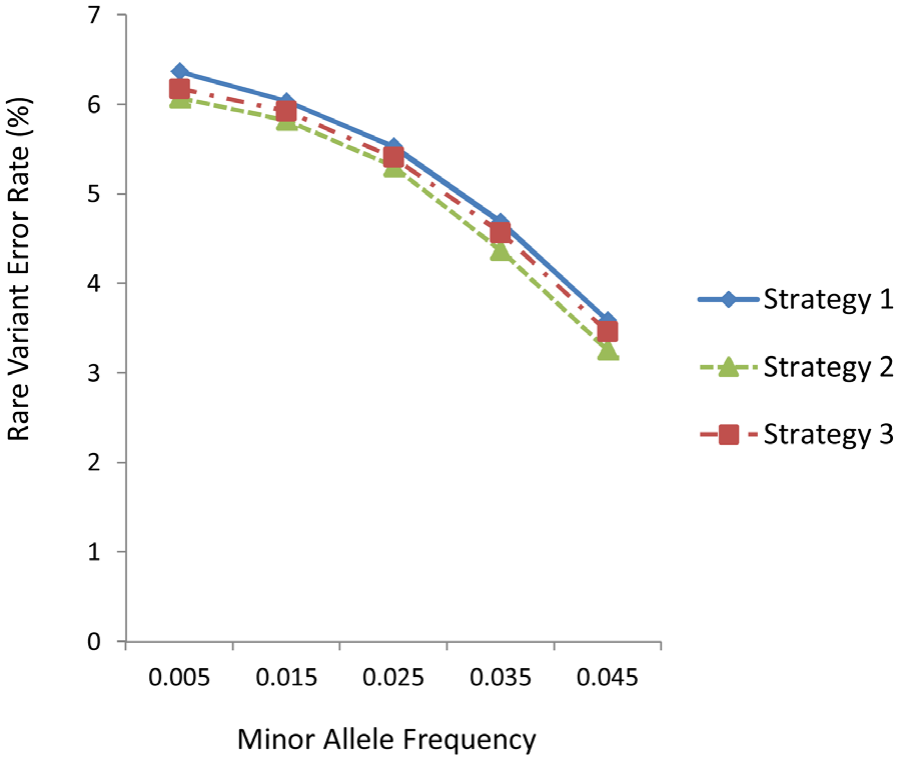
Influence of low frequency rare variant bin on rare variant error rates, under medium LD level and 25% marker density. The results are obtained through the software MACH using empirical data.

The observed effects of various factors, such as LD levels and marker density, on the AERs and RVERs in the empirical data were similar to those of the simulated data. For example, Figure 3A displayed the falling trend of AERs with increasing LD levels. In general, strategy 2 performed better than strategy 1 and strategy 3. Figure 3B showed the falling trend of AERs with increasing marker density, and Figure 3C showed the influence of MAF of un-genotyped markers. Generally, MAF also had similar influence on accuracy with that for the simulated datasets except for the high minor allele frequency of 0.45 (See Table S4, S5, S6 for the detailed estimates used in Figure 3). Similar trends were seen for the other two imputation methods (Figure S2). Figure 4 presented the falling trend of RVERs with increasing MAF of rare variants, and this pattern was similar to that in simulated data sets, too. Again, strategy 2 produced the best performance, and strategy 3 was superior to strategy 1.

## Discussion

Missing genotypes can be imputed because unrelated individuals usually share an expanded haplotype across regions in the whole genome from common ancestors [1], [9]. The reference haplotype sets used for imputation methods are widely used by the human genetics, and their quality will affect the imputation accuracy in GWAS samples. When creating these reference datasets via imputation, it is clear that accuracy should be the most important factor. Next generation sequencing technology will allow researchers to assess many more SNPs. Specifically, 1000 Genomes panels will enable the imputation of many more rare variants with a frequency of 1–5%. Compared to HapMap3, the number of SNPs, haplotypes and populations in 1000 Genomes will increase remarkably. The challenges of imputation analysis will be in using the larger, more diverse set of references available for imputation. In addition, as any haplotype estimates produced from the 1000 Genomes Project data may have more instinctive uncertainty than the HapMap3 haplotypes, owing to the low-coverage sequencing used and the larger number of rare SNPs. As new reference sets with ever larger numbers of variants and haplotypes continue to be made available, GWASs will need to re-impute their datasets from these reference sets.

In this study, we investigated and compared the performance of three imputation strategies on the simulated and empirical data. Using both simulated and empirical data sets, in comparing the different strategies to one another, both strategy 2 and strategy 3 create the better results than the strategy 1, the reason is that both strategies is combing more reference information. More references will present supplementary information and will also create more reliable estimates of measured parameters, resulting in generally decreased error rate. For two combined strategies, strategy 2 produced better results than strategy 3, although most combined method was imputing each other first, and then combined these two references as one reference to impute the validation sample (strategy 3). The reason was that strategy 2 avoided the noise brought about because of repeated imputation (strategy 3), and retained more reliable genotype information from high accuracy reference. Another advantage of the strategy 2 was that it was general enough to be used in order to combine the other re-sequencing data with high coverage, due to avoid imputing reference with each other. Because of this, it was also easy to combine re-sequence data for special region to impute using strategy 2, e.g. Exon, function region. In addition, for our study, validation data and reference data were sampled from the same population, which was the basic assumption for most of the methods studied here. Imputation quality would be reduced with the individuals from a less common ancestry. Importantly, several previous studies have demonstrated the feasibility of using homogeneous samples for reference data [20].

In our research, for rare variants, the results showed that rare variant has higher RVER compared to the other frequency alleles. For the common variants from our study, imputation results were exceedingly accuracy and allow for integration of data sets in meta-analyses. There were some reasons for that. Firstly, rare variants were hard to impute computationally, a variant must be detected several times within its haplotype, due to need high-confidence haplotype information. Secondly, computational approaches cannot impute *de novo* mutations in an individual, unless mutations on the individual's relative are available. Thirdly, it was challenging to distinguish real rare alleles from sequencing errors, particularly when individuals are sequenced at low depth [28]. For solving these issues, sequencing data with high depth can be used with the development of the next generation sequencing technologies. In additional, based our strategies proposed, strategy 2 can increase the rare variant imputation accuracy by incorporating existing high accuracy reference or HapMap3 data into next generation sequencing data. It is also another choice to improve accuracy of the rare variant detection.

Our study provides a description of imputation performance for multiple references under three strategies. Analysis of real data and simulation study show that the strategy 2 (imputes samples by using individual available data sets as independent references and then combines the imputed samples) performs very well compared to the other two strategies. Considering our results, investigators may choose the most appropriate reference population and imputation tool(s) to use based on their specific experimental setting and available computational resources. Given the rapid increase in use of next-generation sequencing technologies, our results should be of value to both empiricists, during experimental design, and to bioinformaticians who seek guidance for selecting appropriate imputation tool(s) and reference populations for data analyses and who attempt improvement of the imputation.

The URLs for software and data presented herein are as follows, the default parameter were used for all the three impute software.

**MACH:** http://www.sph.umich.edu/csg/abecasis/MACH/download/

**IMPUTE2:** http://mathgen.stats.ox.ac.uk/impute/impute_v2.html

**BEAGLE:** http://faculty.washington.edu/browning/BEAGLE/BEAGLE.html

**HAPGEN2:** https://mathgen.stats.ox.ac.uk/genetics_software/hapgen/hapgen2.html

**Hapmap3:** http://hapmap.ncbi.nlm.nih.gov/downloads/phasing/2009-02_phaseIII/HapMap3 r2/

**1000 Genomes:** ftp://ftp-trace.ncbi.nih.gov/1000genomes/ftp/release/20100804/

## Supporting Information

Figure S1 **Effects of LD levels on allele error rates for three strategies with 25% marker density.** (a) The results are based on software Beagle. (b) The results are based on software Impute2 using simulated data.
(TIF)

Figure S2 **Effects of LD levels on allele error rates for three software with 25% marker density.** The results are based on strategy 2 using empirical data.
(TIF)

Table S1 **Effects of LD levels on allele error rates.** The results are based on the simulated data. The values in each cell are mean±SD. The results are presented in Figure 1A in the main text. The data are included here to allow distinction of lines, as certain lines in the figure are close and may be difficult to be distinguished.
(DOC)

Table S2 **Effects of marker density on allele error rates.** The results are based on the simulated data. The values in each cell are mean±SD. The results are presented in Figure 1B in the main text. The data are included here to allow distinction of lines, as certain lines in the figure are close and may be difficult to be distinguished.
(DOC)

Table S3 **Effects of MAF bin of un-genotyped SNPs on allele error rates.** The results are based on the simulated data. The values in each cell are mean±SD. The results are presented in Figure 1C in the main text. The data are included here to allow distinction of lines, as certain lines in the figure are close and may be difficult to be distinguished.
(DOC)

Table S4 **Effects of LD levels on allele error rates.** The results are based on the empirical datasets. The values in each cell are mean±SD. The results are presented in Figure 3A in the main text. The data are included here to allow distinction of lines, as certain lines in the figure are close and may be difficult to be distinguished.
(DOC)

Table S5 **Effects of marker density on allele error rates.** The results are based on the empirical datasets. The values in each cell are mean±SD. The results are presented in Figure 3B in the main text. The data are included here to allow distinction of lines, as certain lines in the figure are close and may be difficult to be distinguished.
(DOC)

Table S6 **Effects of MAF bin of un-genotyped SNPs on allele error rates.** The results are based on the empirical datasets. The values in each cell are mean±SD. The results are presented in Figure 3C in the main text. The data are included here to allow distinction of lines, as certain lines in the figure are close and may be difficult to be distinguished.
(DOC)
