# Supplementary figures and images for: On Combining Reference Data to Improve Imputation Accuracy
Source: PLoS One. 2013 Jan 30;8(1):e55600. doi: 10.1371/journal.pone.0055600 (PMC3559437; doi:10.1371/journal.pone.0055600)

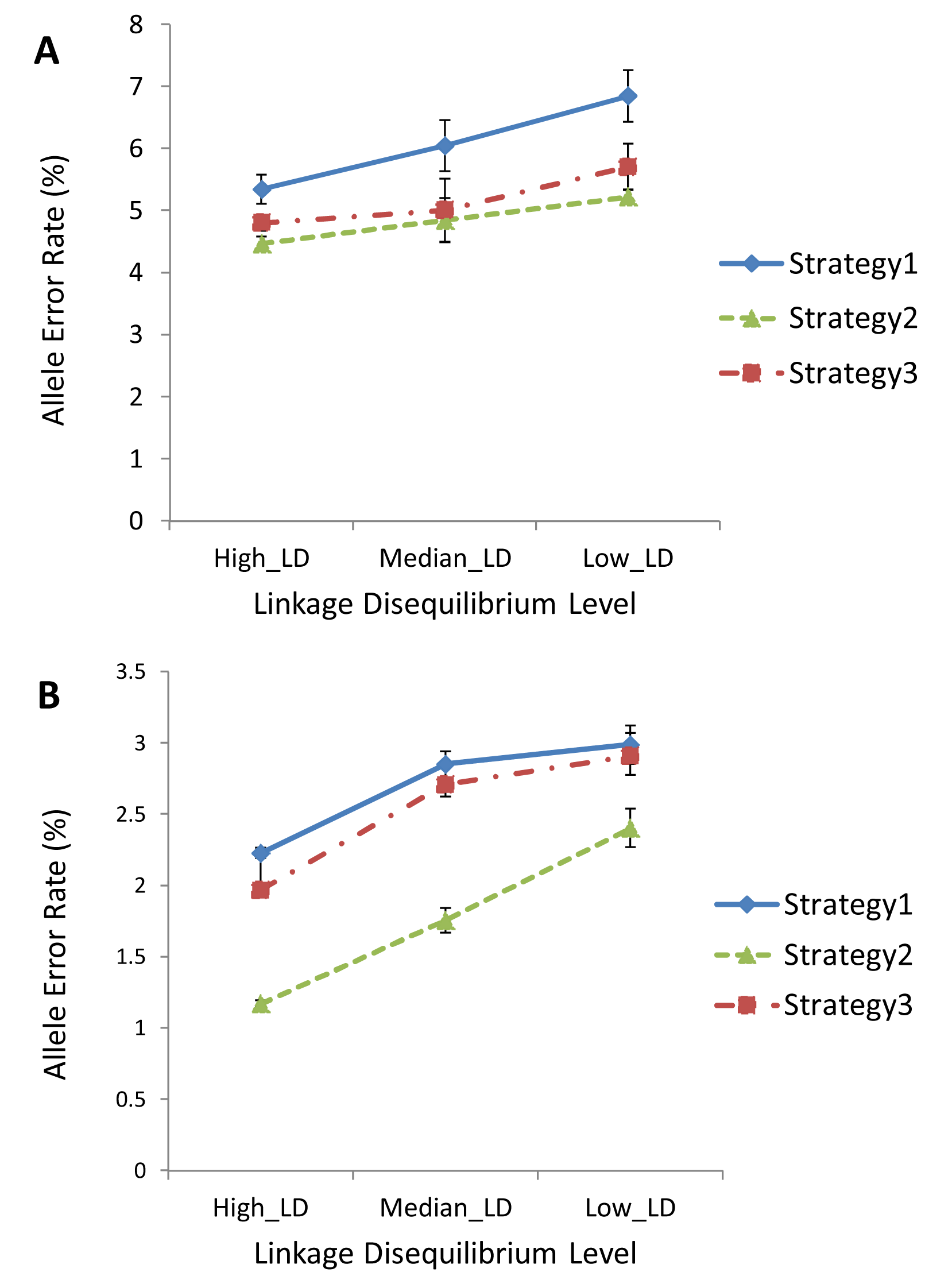

Supplement: Figure S1 — Effects of LD levels on allele error rates for three strategies with 25% marker density. (a) The results are based on software Beagle. (b) The results are based on software Impute2 using simulated data. (TIF) [file pone.0055600.s001.tif]

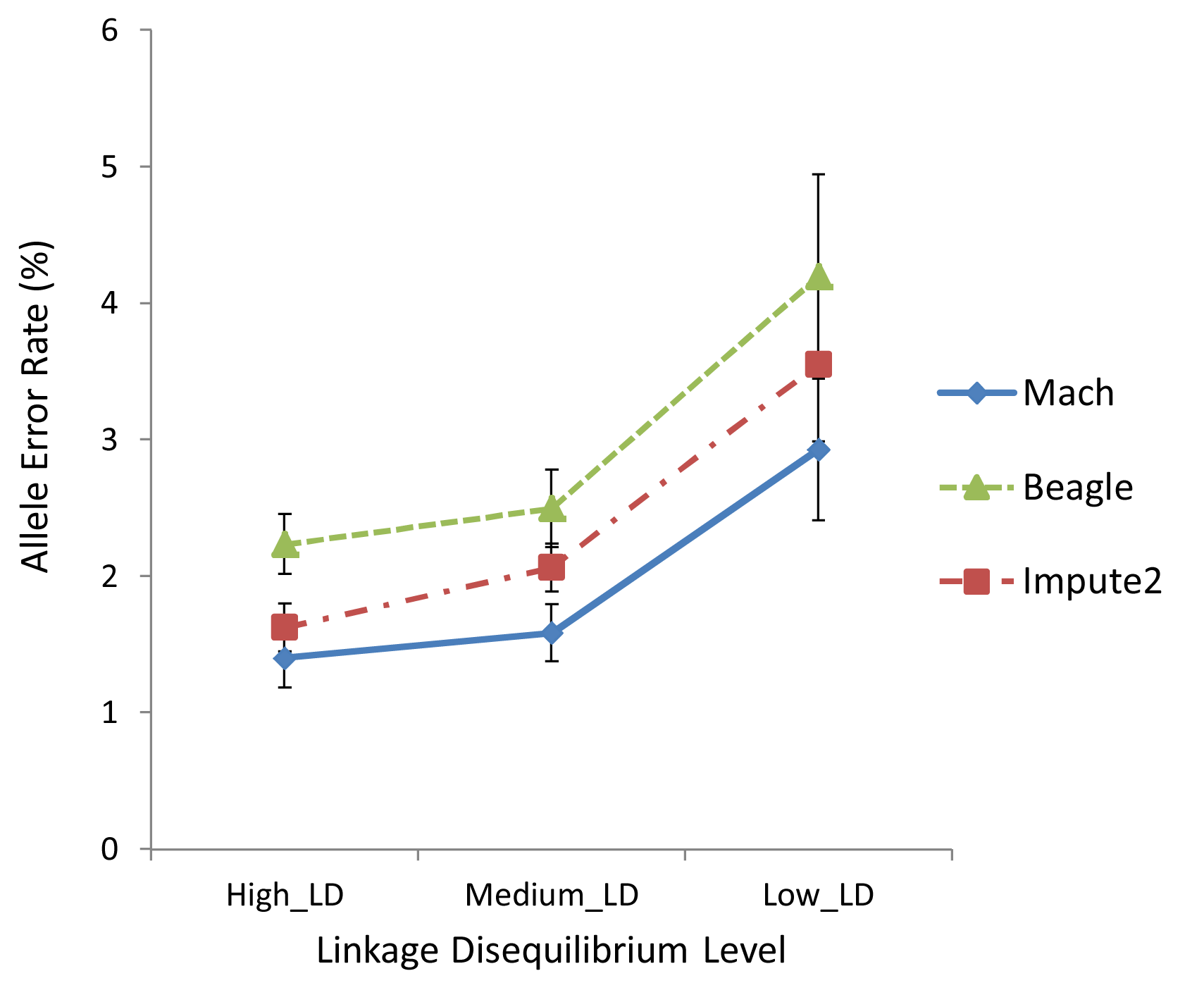

Supplement: Figure S2 — Effects of LD levels on allele error rates for three software with 25% marker density. The results are based on strategy 2 using empirical data. (TIF) [file pone.0055600.s002.tif]
