# Supplementary material for: On Combining Reference Data to Improve Imputation Accuracy
Source: PLoS One. 2013 Jan 30;8(1):e55600. doi: 10.1371/journal.pone.0055600 (PMC3559437; doi:10.1371/journal.pone.0055600)
Supplement: Table S1 — Effects of LD levels on allele error rates. The results are based on the simulated data. The values in each cell are mean±SD. The results are presented in Figure 1A in the main text. The data are included here to allow distinction of lines, as certain lines in the figure are close and may be difficult to be distinguished. (DOC) [file pone.0055600.s003.doc]

**Table S1. Effects of LD levels on allele error rates.**

| Strategy | Linkage Disequilibrium Level | | |
| --- | --- | --- | --- |
|  | High LD | Medium LD | Low LD |
| Strategy1 | 1.91±0.05 | 2.65±0.18 | 2.87±0.08 |
| Strategy2 | 1.09±0.01 | 1.56±0.10 | 1.81±0.15 |
| Strategy3 | 1.73±0.15 | 2.52±0.22 | 2.87±0.11 |

The results are based on the simulated data. The values in each cell are mean±SD. The results are presented in Figure 1A in the main text. The data are included here to allow distinction of lines, as certain lines in the figure are close and may be difficult to be distinguished.
