# Supplementary material for: On Combining Reference Data to Improve Imputation Accuracy
Source: PLoS One. 2013 Jan 30;8(1):e55600. doi: 10.1371/journal.pone.0055600 (PMC3559437; doi:10.1371/journal.pone.0055600)
Supplement: Table S3 — Effects of MAF bin of un-genotyped SNPs on allele error rates. The results are based on the simulated data. The values in each cell are mean±SD. The results are presented in Figure 1C in the main text. The data are included here to allow distinction of lines, as certain lines in the figure are close and may be difficult to be distinguished. (DOC) [file pone.0055600.s005.doc]

**Table S3. Effects of MAF bin of un-genotyped SNPs on allele error rates.**

| Strategy | Minor Allele Frequency | | | | |
| --- | --- | --- | --- | --- | --- |
|  | 0.05 | 0.15 | 0.25 | 0.35 | 0.45 |
| Strategy1 | 1.65 | 2.81 | 3.84 | 4.52 | 4.98 |
| Strategy2 | 0.90 | 1.46 | 1.92 | 2.37 | 2.35 |
| Strategy3 | 1.57 | 2.78 | 3.60 | 4.33 | 4.81 |

The results are based on the simulated data. The values in each cell are mean±SD. The results are presented in Figure 1C in the main text. The data are included here to allow distinction of lines, as certain lines in the figure are close and may be difficult to be distinguished.
